# An exploration of mechanism of high quality and yield of *Gastrodia elata* Bl*. f. glauca* by the isolation, identification and evaluation of *Armillaria*

**DOI:** 10.1186/s12870-022-04007-8

**Published:** 2022-12-30

**Authors:** En Yu, Yugang Gao, Yaqi Li, Pu Zang, Yan Zhao, Zhongmei He

**Affiliations:** College of Chinese Medicinal Materials, Jilin Agricultural University, Changchun, 130118 China

**Keywords:** *Gastrodia elata* Bl. *f. glauca* S. chow, *Armillaria*, Separation and identification, Nutrition elements, Microbial diversity

## Abstract

**Background:**

*Gastrodia elata* Bl. *f. glauca*, a perennial herb of *G.elata* Bl. in Orchidaceae, is one of the most valuable traditional Chinese medicines. *G. elata* Bl. is a chlorophyll-free myco-heterotrophic plant, which must rely on the symbiotic growth of *Armillaria*, but not all *Armillaria* strains can play the symbiotic role. Additionally, *Armillaria* is easy to degenerate after multiple generations, and the compatibility between the strains from other areas and *G. elata* Bl. *f. glauca* in Changbai Mountain is unstable. Therefore, it is incredibly significant to isolate, identify and screen the symbiotic *Armillaria* suitable for the growth of *G. elata* Bl. *f. glauca* in Changbai Mountain, and to explore the mechanism by which *Armillaria* improves the production performance of *G. elata* Bl. *f. glauca*.

**Results:**

Firstly, *G. elata* Bl. *f. glauca* tubers, and rhizomorphs and fruiting bodies of *Armillaria* were used for the isolation and identification of *Armillaria*. Five *Armillaria* isolates were obtained in our laboratory and named: JMG, JMA, JMB, JMC and JMD. Secondly, *Armillaria* was selected based on the yield and the effective component content of *G. elata* Bl. *f. glauca*. It was concluded that the yield and quality of *G. elata* Bl. *f. glauca* co-planted with JMG is the highest. Finally, the mechanism of its high quality and yield was explored by investigating the effects of different *Armillaria* strains on the soil, its nutrition element contents and the soil microbial diversity around *G. elata* Bl. *f. glauca* in Changbai Mountain.

**Conclusions:**

Compared with commercial strains, JMG significantly increased the content of Na, Al, Si, Mn, Fe, Zn, Rb and the absorption of C, Na, Mg, Ca, Cr, Cu, Zn and Rb in *G. elata* Bl. *f. glauca*; it improved the composition, diversity and metabolic functions of soil microbial communities around *G. elata* Bl. *f. glauca* at phylum, class and genus levels; it markedly increased the relative abundance of bacteria such as *Chthoniobacter* and *Armillaria* in the dominant populations, and enhanced such functions as Cell motility, amino acid metabolism and Lipid metabolism; it dramatically decreased the relative abundance of *Bryobacter* and other fungi in the dominant populations, and reduced such functions as microbial energy metabolism, translation and carbohydrate metabolism. This is the main reason why excellent *Armillaria* strains promote the high quality and yield of *G. elata* Bl. *f. glauca* in Changbai Mountain.

**Supplementary Information:**

The online version contains supplementary material available at 10.1186/s12870-022-04007-8.

## Introduction

*Gastrodia elata* Bl. *f. glauca* belongs to a variety of *G. elata* Bl., a perennial herb in Orchidaceae. *G.elata* Bl. is also called as Tianma in Chinese. It contains such active ingredients as gastrodin, p-hydroxybenzyl alcohol, balisonoside A, parisenoside B, balisonoside C, balisonoside E [1]. It has the efficacy of suppressing hyperactive liver for calming endogenous wind, relieving pain and spasmolysis, calming lung qi and liver-yang, dispelling wind, removing blood stasis and dredging collaterals [1]. The results of modern pharmacological clinical research have showed that *G. elata* Bl. related drugs can be widely used to reduce chronic hypertension [2], increase cardiac and cerebral blood flow [3], modulate the nervous system [4], and treat such disease as chronic depression [5], and Alzheimer’s disease [6]. The demand for *G. elata* Bl. is growing rapidly at home and abroad, especially the high-quality *G. elata* Bl. *f. glauca* in Changbai Mountain is in short supply [7]. However, the wild *G. elata* Bl. is scarce and artificial plantation is required for its supply.

The growth and development of *G. elata* Bl., as one of the heterotrophic plants without chlorophyll, requires symbiosis with *Armillaria* [8–10], which has a significant impact on the yield and quality of *G. elata* Bl.. The isolation and identification of excellent *Armillaria* strains are of great significance [11]. *Armillaria* is prone to species degradation during multi-generation reproduction, that is, after repeated asexual reproduction, the quality of the offspring of the strain decreases, and the ability to provide nutrients for *G. elata* Bl. decreases. Thus, it requires continuous renewal.

*Armillaria* (Fr.) *Staude* belongs to *Basidiomycota* [12–14]. It is distributed on all continents of the world, and in China, it is mainly distributed in Jilin, Heilongjiang and Inner Mongolia and other provinces [15–17]. At present, over 40 species of *Armillaria* have been described in the world, some of which are serious tree pathogens, some have medicinal value, and some can coexist with *G. elata* Bl. for its cultivation and production [18–20]. There are 15 *Armillaria* species in China, of which eight are endemic to China [21]. In the 1960s, Xu Jintang used the culture method of Armillaria cords, fresh wood segments and *G. elata* Bl. seeds to make *G. elata* Bl. wild and domesticated. In Changbai Mountain, Zhaotong in Yunnan, and Guizhou, a variety of *Armillaria* species that can coexist with *G. elata* Bl. have been identified, including *A. mellea*, *A. gallica*, *A. sinapina* and *A. ce*pistipes. Seven phylogenetic branches can coexist with *G. elata* Bl. [21–26]. New *Armillaria* species in China are constantly being discovered.

As mentioned above, not all *Armillaria* can coexist with *G. elata* Bl.. There are few *Armillaria* species isolated and identified in Changbai Mountain, and fewer strains can be available for symbiosis with *G. elata* Bl. there [27]. The unstable compatibility between strains from other areas and *G. elata* Bl. *f. glauca* affects its yield [28]. It is vital to isolate, identify and screen symbiotic *Armillaria*, which is beneficial to the growth of *G. elata* Bl. *f. glauca*. Studies have shown that many orchid mycorrhizal fungi can promote nutrient absorption in the plant rhizosphere [29]. Meanwhile, they also affect changes in microbial community structures and their functions in rhizosphere soil [30–32]_._ However, the effect of symbiotic fungus *Armillaria* on the absorption of nutrition elements by *G. elata* Bl. [33]. And the ecological relationship as well as nutrient cycles among *G. elata* Bl., *Armillaria*, soil and soil microorganisms remain unknown [34, 35]. Therefore, it is of great significance for its cultivation to explore the mechanism by which the excellent *Armillaria* can improve the production performance of *G. elata* Bl. *f. glauca* [36]*.* In this study, *Armillaria* was first isolated and identified by using *G. elata* Bl. *f. glauca* tubers, rhizomorphs and fruiting bodies of *Armillaria* [37]. Then, *Armillaria* was selected according to the yield and active component content of *G. elata* Bl. *f. glauca*. Finally, the effects of different strains on the nutrition element content and microbial diversity in the soil around *G. elata* Bl. *f. glauca* were investigated to explore the mechanism of high quality and yield of *G. elata* Bl. *f. glauca* co-planted with *Armillaria* [38, 39].

## Results

### Isolation and identification of *Armillaria* from *G. elata* Bl. *f. glauca*

**Fig. 1:**
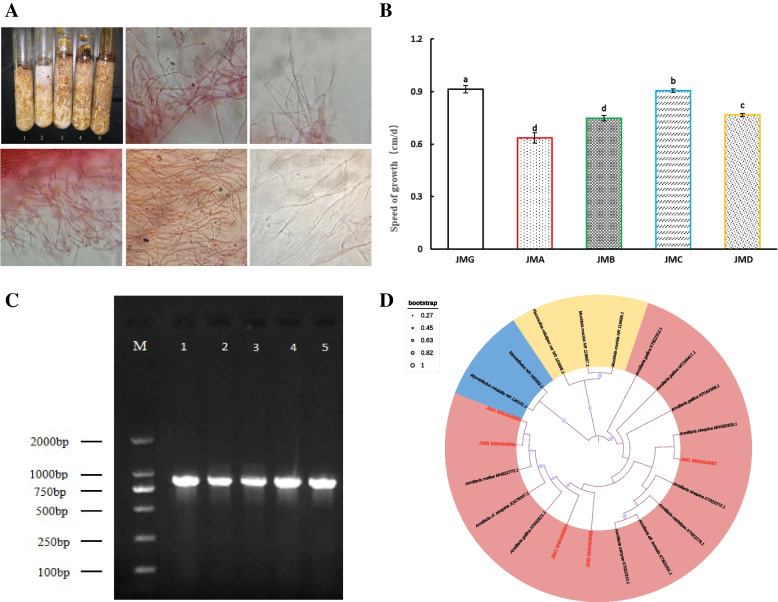
Isolation and identification of isolates. **A** Mycelial morphology and microscopically observed features of five isolates on corn-wheat bran medium at 25 °C after seven days. (1: JMG; 2: JMA; 3: JMB; 4: JMC; 5: JMD). **B** Comparison of mycelial growth rate of five isolates (Lower-case letters indicate significant differences at *p* < 0.05). **C** The electrophoresis of PCR products of five *Armillaria* isolates (M:DNA molecular weight standard, 1: JMG; 2: JMA; 3: JMB; 4: JMC; 5: JMD). **D** Phylogenetic analysis of five isolates based on the internal transcribed spacer-5.8S sequences. The size of the circle represents bootstrp, the larger the circle, the higher the boot, the smaller the circle, the lower the reliability. The phylogenetic tree was constructed using the neighbor-joining method (1000 bootstrap replications). The accession numbers for the sequences retrieved from the GenBank database are listed in Table S4

A total of 11 *Armillaria* isolates were isolated, including four from rhizomorphs, and four from fruiting bodies of *Armillaria,* and three from *G. elata* Bl. *f. glauca* tubers. During the passaging process, the isolates with vigorous mycelial growth were screened. Finally, five *Armillaria* isolates were selected for morphological and molecular identification in our laboratory and named JMG,JMA,JMC,JMD and JMG, respectively. Among them, JMG and JMA were isolated from rhizomorphs, JMB and JMC from fruiting body tissue, and JMD from *G. elata* Bl. *f. glauca* tubers. The morphological characteristics of the mycelium in the five *Armillaria* isolates were different. Among them, the hyphae of the isolates JMG, JMC and JMD spores germinate faster compared with JMA and JMB, and the germination time of the former and later was 5–6 and 6-7 days, respectively. Different *Armillaria* isolates were ranked as follows according to mycelial growth rates from fast to slow: JMG > JMC > JMD > JMB > JMA (Fig. 1B, Table S1). The comparison of microscopic characteristics showed that JMG and JMC had dense hyphae and good growth conditions, while JMA, JMB and JMD had sparse hyphae and relatively poor growth conditions (Fig. 1A). This indicated that there are indeed morphological differences between these five isolates.

PCR amplification of the ITS rDNA region yielded five single bands ranging in size from 750 to 1000 bp (Fig. 1C). Through The Basic Local Alignment Search Tool (BLAST) homology comparison, it was found that the base sequences of the five *Armillaria* isolates shared over 98% homology with registered *Armillaria* genes in NCBI database (Accession Nos. MW404994, MW404995, MW404996, MW404997and MW404998), and that the same base sequence coverage was more than 97%. BLAST homology comparison showed that the five isolates shared more than 97% homology with *A. gallica* and *A. sinapina* (Table S2). The phylogenetic tree showed that the five isolates were clustered in three main clades, of which JMG (MW404994) and JMD (MW404998) had the shortest clade with *A. gallica*, JMA (MW404995) and JMB (MW404996) had the shortest branches with *A. mellea*，and JMC (MW404997) had the shortest branches with *A. sinapina* (Fig. 1D).

### Optimization of *Armillaria* from *G. elata* Bl. *f. glauca*

**Fig. 2:**
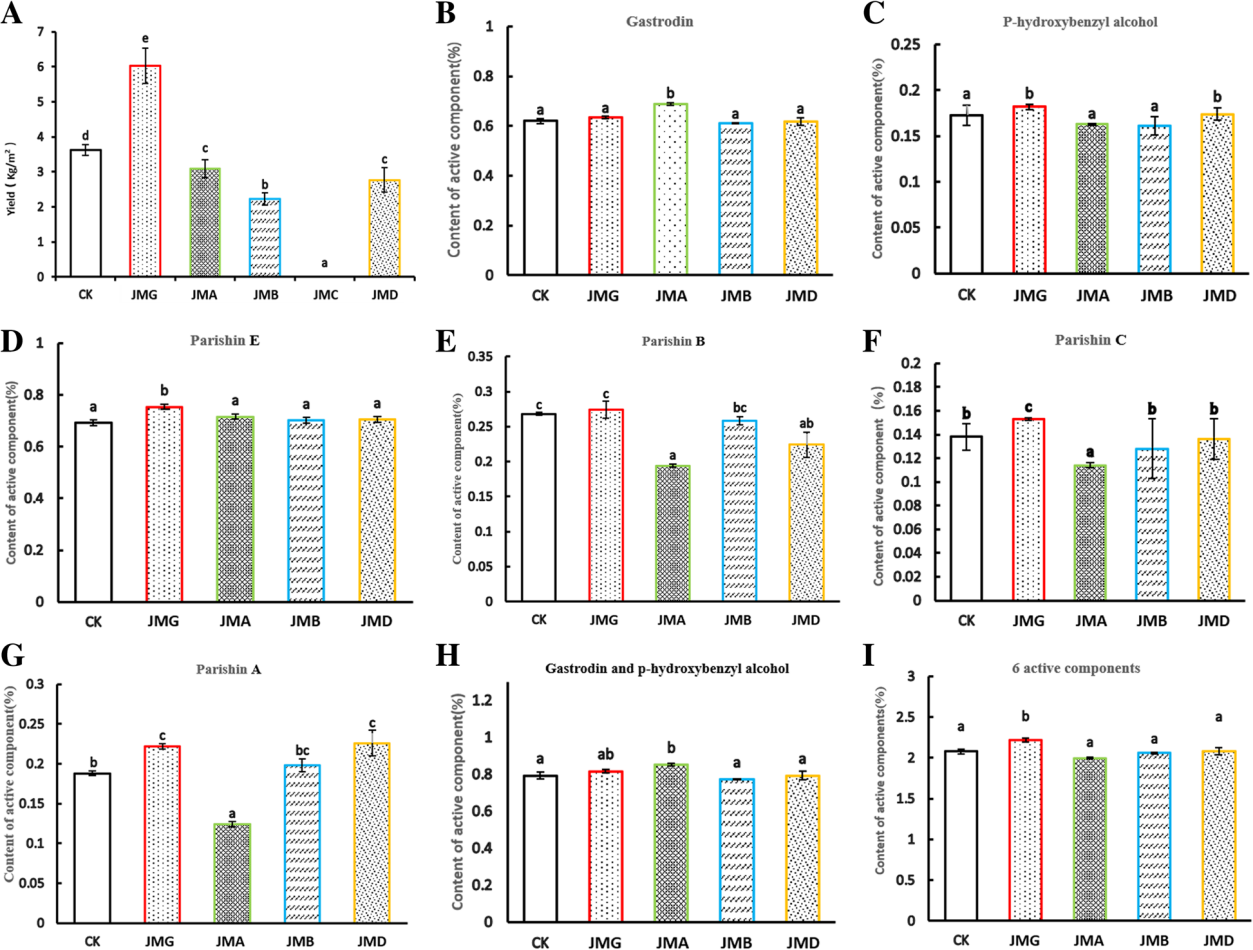
Effects of different *Armillaria* isolates on the yield and active components of *Gastrodia elata* Bl. *f. glauca.* **A** Effects of different *Armillaria* isolates on the yield of *G. elata* Bl. *f. glauca.* **B-I** Contents of active components of different *Armillaria* isolates with *G. elata* Bl. *f. glauca*. CK: The commercial *Armillaria* strain; JMG, JMA, JMB, JMC, JMD: five isolates of *Armillaria*. Lower-case letters indicate significant differences at p < 0.05

The results of field experiments showed that four *Armillaria* isolates could promote the growth of *G. elata* Bl. *f. glauca* tubers except JMC, and that different isolates had significant effects on the yield of *G. elata* Bl. *f. glauca* (*P* < 0.05). The yield of *G. elata* Bl. *f. glauca* co-planted with each isolate was all significantly higher, compared with the commercial strain control check (CK) (*P* < 0.05). The yield of *G. elata* Bl.*f. glauca* co-planted with the commercial strain CK was significantly higher, compared with JMA and JMD (P < 0.05), and the yield of *G. elata* Bl. *f. glauca* co-planted with JMA and JMD was both markedly higher, compared with JMB (*P* < 0.05) (Fig. 2A, Table S3).

*G. elata* Bl. *f. glauca* co-planted with each isolate all contained six active components, gastrodin, p-hydroxybenzyl alcohol, parishin E, parishin B, parishin C and parishin A, respectively (Fig. S1A-F). There were significant differences in the content of the six active components among *G. elata* Bl. co-planted with different *Armillaria isolates* (*P* < 0.05). The gastrodin content in the JMA group was significantly higher than that in the other groups, respectively, but with no significant difference among the other groups (Fig. 2B). The content of p-hydroxybenzyl alcohol in JMG and JMD groups were significantly higher than that in the other groups, respectively, but with no significant difference among the other groups (Fig. 2C). The content of parishin E in JMG group was significantly higher than that in the other groups, respectively (*P* < 0.05), but with no significant difference among the other groups (Fig. 2D). The content of parishin B in JMG and CK groups was significantly higher than that in JMD group, respectively (*P* < 0.05), and the content of parishin B in JMD and JMB groups were significantly higher than that in JMA group, respectively (*P* < 0.05) (Fig. 2E). The content of parishin C in JMG group was significantly higher than that in CK, JMB and JMD groups, respectively and the content of parishin C in CK, JMB and JMD groups was significantly higher than that in JMA group, respectively. There was no significant difference among CK, JMB and JMD groups (Fig. 2F). The content of parishin A in JMG and JMD groups was significantly higher than that in CK group, respectively, and the content of parishin A in CK and JMB groups was significantly higher than that in JMA group (*P* < 0.05). There was no significant difference among the other groups (Fig. 2G). The additive value of gastrodin and p-hydroxybenzyl alcohol in the JMA group was significantly higher than that in CK, JMB and JMD groups, respectively (*P* < 0.05), but with no significant difference among the other groups (Fig. 2H). The sum value of six active ingredients in the JMG group was significantly higher than that in the other groups (*P* < 0.05), but with no significant difference among the other groups (Fig. 2I). It can be concluded that both the yield and effective component content of *G. elata* Bl. *f. glauca* co-planted with JMG are better compared with the other isolates. Thus, JMG is more suitable for the cultivation of *G. elata* Bl. *f. glauca*.

### Analysis of absorption Laws of nutrition elements in *G. elata* Bl. *f. glauca* co-planted with JMG

The inorganic elements in the surrounding soil of *G. elata* Bl. *f. glauca* and its tubers under the symbiosis of it with different *Armillaria* (the Commercial strain CK, JMG, respectively) were detected by ICP-MS. More than 150 inorganic elements were detected, among which there were 28 related to plant growth. There were 14 chemical elements in *G. elata* Bl. *f. glauca* (C, N, Na, Mg, Al, Si, P, S, K, Ca, Mn, Fe, Zn, Rb). The chemical elements (Na, Al, Si, Mn, Fe, Zn, Rb) of *G. elata* Bl. *f. glauca* co-planted with JMG were higher, compared with commercial *Armillaria* strains. There were significant differences in 10 soil chemical elements (C, N, Na, Mg, Al, Si, S, K, Ca, Fe). Among them, the soil chemical elements of *G. elata* Bl. *f. glauca* co-planted with JMG were all higher, compared with commercial *Armillaria* strains. The enrichment coefficients of 10 elements (B, C, Na, Mg, P, Ca, Cr, Cu, Zn, Rb) were significantly different. Among them, the enrichment elements (C, Na, Mg, Ca, Cr, Cu, Zn, Rb) of *G. elata* Bl. *f. glauca* co-planted with JMG were all higher, compared with commercial *Armillaria* strains. It can be seen that JMG significantly increased the content of chemical elements (Na, Al, Si, Mn, Fe, Zn and Rb) and the absorption of chemical elements (C, Na, Mg, Ca, Cr, Cu, Zn and Rb) in *G. elata* Bl. *f. glauca* co-planted with it. This is one of the reasons for high quality and yield of *G. elata* Bl. *f. glauca* (Table S4).

### Microbial sequence analysis of the soil around *G. elata* Bl. *f. glauca* co-planted with JMG

**Fig. 3:**
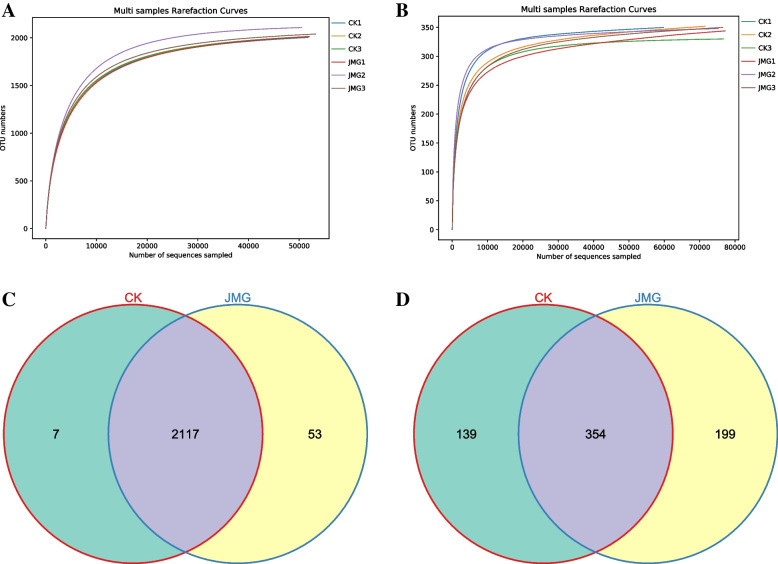
Sequence analysis of soil microorganisms around *Gastrodia elata* Bl. *f. glauca* in two symbiotic of it with CK and JMG. The digital number represented three biological replicates for each sample. Bacteria (**A**) and fungi (**B**) dilution curves of soil samples. OTU count of bacteria (**C**) and fungi (**D**) in soil samples. CK: The commercial *Armillaria* strain

Sequence analysis showed that the number of high-quality sequences of fungi was 453,881, and that the number of high-quality sequences of bacteria was 475,153. With the number of read sequences increasing, the number of OTUs increased rapidly and then slowly, and when the number of sequences exceeded 10,000, the number of OTUs tended to flatten, indicating that the sample sequences can cover most of the species in soil samples (Fig. 3A and B). The V-enn diagram of each sample showed that the OTU number in CK and JMG groups was 2124 and 2170 respectively, with a total of 2117, 7 unique bacteria in the CK group and 53 unique bacteria in the JMG group (Fig. 3C). The OTU number in CK and JMG groups was 493,553, respectively. There were 354 fungi in the CK group and 199 fungi in the JMG group (Fig. 3D). It can be seen that the sample sequences in both JMG and CK groups covered most of the same species, but the OUT number in the JMG group was larger, compared with the CK one, and the OTU number of bacteria and fungi unique to the JMG group was larger, compared with the CK one. The results showed that the co-planting of different *Armillaria* isolates with *G. elata* Bl. *f. glauca* had significant effects on the soil microbiota around its tubers.

### The composition of soil microbial communities around *G. elata* Bl. *f. glauca* co-planted with JMG

**Fig. 4:**
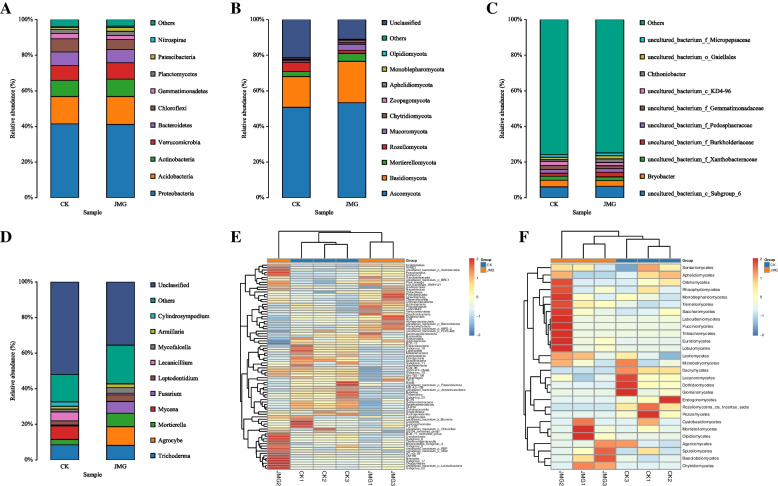
The composition of soil microbial community around *Gastrodia elata* Bl. *f. glauca* in two symbiotic of it with JMG and CK. The digital number represented three biological replicates for each sample. Relative abundance of bacteria (**A**) and fungi (**B**) in each sample at the phylum level. Relative abundance of bacteria (**C**) and fungi (**D**) in each sample at the genus level. Relative abundance of bacteria (**E**) and fungi (**F**) in each sample at the class level. CK: The commercial *Armillaria* strain

Firstly, it was observed that the relative abundance of soil bacteria and fungi around *G. elata* Bl. *f. glauca* under the symbiosis of it with different *Armillaria* strains at the phylum level. Among them, the dominant bacteria in the soil around *G. elata* Bl. *f. glauca* tubers were mainly from *Proteobacteria*, *Acidobacteria*, *Actinobacteria*, *Verrucomicrobia*, *Bacteroidetes*, *Chloroflexi*, *Gemmatimonadetes*, *Planctomycetes*, *Patescibacteria* and *Nitrospirae*. The relative abundance of *Acidobacteria*, *Verrucomicrobia*, *Planctomycetes* and *Patescibacteria* increased by 0.77, 0.73, 0.12 and 1.02% respectively in the soil around *G. elata* Bl. *f. glauca* co-planted with JMG, compared with the commercial strain CK, while the abundance of *Chloroflexi* and *Gemmatimonadetes* decreased by 1.79 and 0.75%, respectively (Fig. 4A). The dominant fungi in the soil around *G. elata* Bl. *f. glauca* tubers mainly came from *Basediomycota*, *Ascomycota*, *Mucoromycota*, *Mortierellomycota*, *Rozellomycota*, *Chytridiomycota*, *Zoopagomycota*, *Aphelidiomycota*, *Monoblepharomycota* and *Olpidiomycota*. The relative abundance of *Basidiomycetes*, *Ascomycetes*, *Mucoromycota* and *Mortierellomycota* increased by 6.05, 2.52, 2.48 and 1.67% respectively in the soil around *G. elata* Bl. *f. glauca* tubers co-planted with JMG, compared with the commercial strain CK, while the relative abundance of *Rozellomycota* decreased by 3.58% (Fig. 4B).

Secondly, the differences in the relative abundance of soil bacteria and fungi around *G. elata* Bl. *f. glauca* under the symbiosis of it with different *Armillaria* strains at the genus level were observed. Among them, the dominant bacteria in the soil around *G. elata* Bl. *f. glauca* showed a variety of uncultured-bacterium. The relative abundance of *Chthoniobacter* and *uncultured_bacterium_f_Burkholderiaceae* in the soil around *G. elata* Bl. *f. glauca c*o-planted with JMG increased by 0.94 and 0.88% respectively, compared with the commercial strain CK, while the relative abundance of *Bryobacter* and *uncultured_bacterium_f_Gemmatimonadaceae* decreased by 0.62 and 0.61%, respectively (Fig. 4C). The dominant fungi in the soil around *G. elata* Bl. *f. glauca* mainly came from *Trichoderma*, *Agrocybe*, *Mortierella*, *Mycena*, *Fusarium*, *Leptodontidium*, *Lecanicillium*, *Mycofalcella*, *Armillaria* and *Cylindrosympodium*. The relative abundance of *Armillaria, Mortierella, Fusarium and Agrocybe* in the soil around *G. elata* Bl. *f. glauca* co-planted with JMG increased by 3.00, 1.53, 0.46 and 0.01%, respectively, compared with the commercial strain CK, while the relative abundance of *Lecanicillium*, *Cylindrosympodium* and *Mycena* decreased by 0.95, 0.03 and 0.01%, respectively (Fig. 4D). The results showed that JMG improved the composition of bacterial and fungal communities in the soil around *G. elata* Bl. *f. glauca.*

Finally, the cluster differences in soil bacterial and fungal species abundance around *G. elata* Bl. *f. glauca* under the symbiosis of it with different *Armillaria* strains at the class level were investigated. The soil bacteria around *G. elata* Bl. *f. glauca* co-planted with JMG were clustered into *Acidimicrobiia*, *Acidobacteria*, *Oxyphotobacteria*, *Alpha proteobacterium*, *Fimbriimonadia*, *Thermoleophilia*, *Coriobacteriia* and *Nitrospirae* (Fig. 4E). The soil bacteria around *G. elata* Bl. *f. glauca* co-planted with the commercial strain CK were clustered into *Anaerolineae*, *Bacilli*, *Armatimonadia*, *Clostridia*, *Sericytochromatia* and *Ktedonobacteria*. Meanwhile, the soil fungi around *G. elata* Bl. *f. glauca* co-planted with JMG were clustered into *Orbiliomycetes*, *Rhizophydiomycetes*, *Saccharomycetes*, *Laboulbeniomycetes*, *Pucciniomycetes*, *Tritirachiomycetes*, *Tremellomycetes*, *Lobulomycetes*, *Eurotiomycetes*, *Monoblepharidomycetes*, *Mortierellomycetes*, *Cystobasidlomycetes* and *Olpidiomycetes*. The soil fungi around *G. elata* Bl. *f. glauca* co-planted with commercial strains were clustered into *Dacrymycetes*, *Lecanoromycetes*, *Dothideomycetes*, *Glomomycetes and Pezizomycetes* (Fig. 4F). There were significant differences in clustering between the JMG and CK groups at the class level. The results showed that JMG improved the composition of bacterial and fungal communities in the soil around *G. elata* Bl. *f. glauca* at the phylum, class and genus levels. This may be one of the reasons why JMG promoted the absorption of soil nutrients by *G. elata* Bl. *f. glauca* and increased its yield.

### Alpha and Beta diversity of soil microbial communities around *G. elata* Bl. *f. glauca* co-planted with JMG

**Fig. 5:**
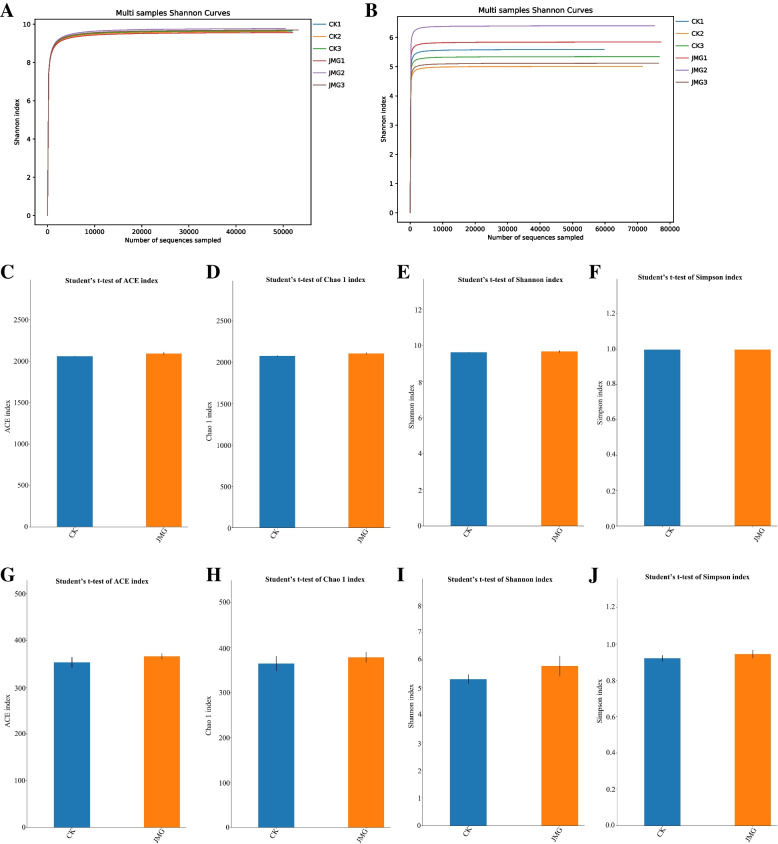
Analysis of soil microbial diversity around *Gastrodia elata* Bl. *f. glauca* in two symbiotic relationship of it with JMG and CK. The digital number represented three biological replicates for each sample. Shannon diversity index dilution curve of bacteria (**A**) and fungi (**B**) in soil samples. Histogram of differences between groups of soil bacterial diversity Ace index (**C**), Chao1 index (**D**), Shannon index (**E**) and Simpson index (**F**). Histogram of differences between groups of soil fungal diversity Ace index (**G**), Chao1 index (**H**), Shannon index (**I**) and Simpson index (**J**). CK: The commercial *Armillaria* strain. Alpha Diversity Index statistics from Table S6

Although the above results demonstrated that JMG affects the composition of soil microbial communities around *G. elata* Bl. *f. glauca*, its effects on the community diversity have yet been unclear. In order to further investigate whether JMG affects the soil microbial community diversity around the *G. elata* Bl. *f. glauca*, the soil microbial α-diversity around *G. elata* Bl. *f. glauca* tubers was firstly analyzed. In the 0–5000 range, Shannon index increased sharply with the continuous increase of sequence number. When the number of sequences exceeded 5000, the curve tended to flatten, and Shannon index would no longer increase with the continuous increase of sequence number (Fig. 5A and B). The number of sequencing data was large enough to cover the information related to most microorganisms and their species. It was found that there was no significant change in soil bacterial α-diversity around *G. elata* Bl. *f. glauca* in the JMG and CK groups. (Fig. 5C-F), but the the Ace, Chao1, Shannon and Simpson indexes ones of fungi in the soil around *G. elata* Bl. *f. glauca* co-planted with JMG were higher, compared with the CK (Fig. 5G-J). The data coverage of the sequencing library of the samples in this study exceeded 99.9 and 98.5% respectively, indicating that the sample sequencing results fully reflect the real situation of various microorganisms in each sample.

**Fig. 6:**
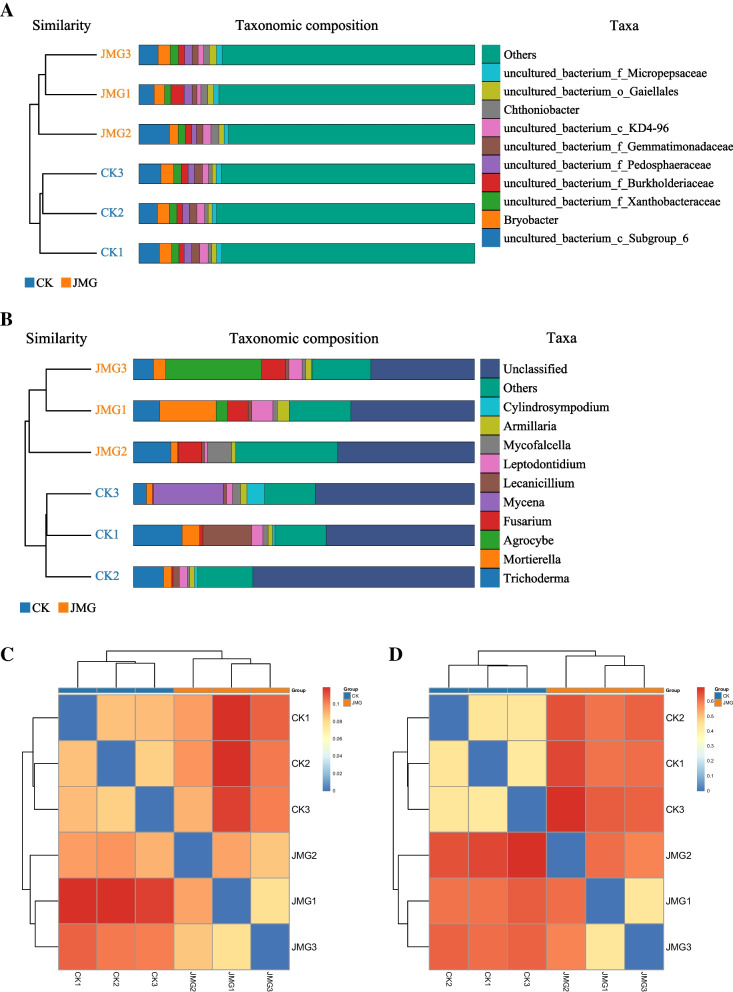
Analysis of soil microbial diversity around *Gastrodia elata* Bl*. f. glauca* in two symbiotic of it with JMG and CK. The digital number represented three biological replicates for each sample. UPGMA Analysis of bacteria (**A**) and fungi (**B**) in soil samples. Cluster heatmaps of bacteria (**C**) and fungi (**D**) in soil sample. CK: The commercial *Armillaria* strain

Secondly, the soil microbial β-diversity around *G. elata* Bl. *f. glauca* tubers was analyzed. It was found that the soil microbial population around *G. elata* Bl. *f. glauca* under the symbiosis of it with JMG and the commercial strain CK was clustered on their own branches (Fig. 6A and B). The clustering heatmap showed that the rank abundance of both soil bacterial and fungal samples around *G. elata* Bl. *f. glauca* co-planted under the symbiosis of it with the commercial strain CK and JMG were both clustered into class1 (Fig. 6C and D). The results showed that there were different microbial diversities in the soil around *G. elata* Bl. *f. glauca* under the symbiosis of it with different *Armillaria* strains. The symbiosis of JMG and *G. elata* Bl. *f. glauca* increased the microbial diversity in the soil around *G. elata* Bl. *f. glauca*, which may be another mechanism by which JMG promoted the soil nutrient absorption by *G. elata* Bl. *f. glauca* and increased its yield.

### The analysis of soil microbial differences around *G. elata* Bl. *f. glauca* co-planted with JMG

**Fig. 7:**
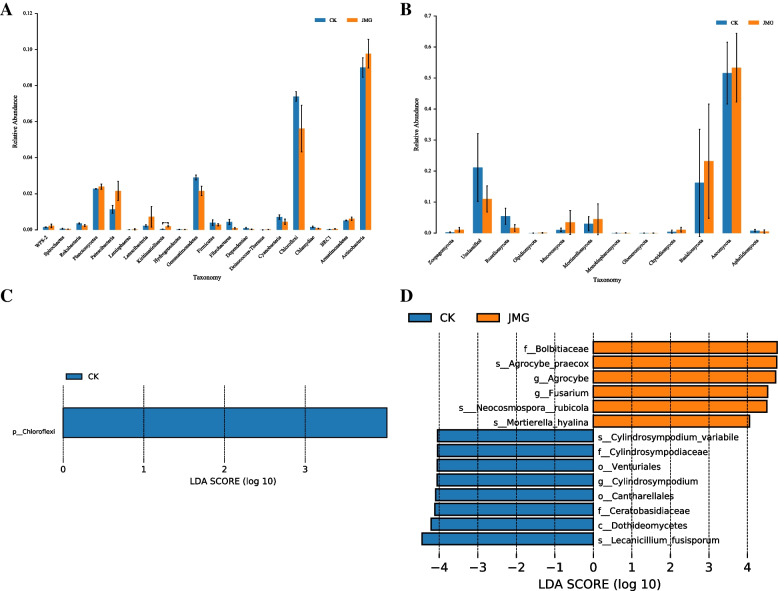
Difference analysis of soil microbiome around *Gastrodia elata* Bl. *f. glauca* between two symbiotic of it with JMG and CK. ANOVA analysis of bacteria (**A**) and fungi (**B**) in soil samples. Distribution histogram of LDA values of bacteria (**A**) and fungi (**B**) in soil samples. CK: The commercial *Armillaria* strain

Based on the significant differences in the soil microbial composition and diversity around *G. elata* Bl. *f. glauca* under the symbiosis of it with JMG and the commercial strain CK, the differences between the two groups of samples were further investigated. First, through ANOVA analysis, there were significant differences among *Actinobacteria*, *Chloroflexi*, *Gemmatimonadetes*, *Planctomycetes*, *Patescibacteria* and *Cyanobacteria* at the level of bacteria (*P* < 0.05). Among them, the relative abundance of *Actinobacteria*, *Patescibacteria* and *Planctomycetes* in the soil around *G. elata* Bl. *f. glauca* co-planted with JMG was higher (Fig. 7A). At the fungal phylum level, there were significant differences among *Ascomycota*, *Basediomycota*, *Mortierellomycota*, *Rozellomycota*, *Mucoromycota*, *Chytridiomycota* and *Zoopagomycota* in different groups. Among them, the relative abundance of *Mucoromycota*, *Mortierellomycota*, *Chytridiomycota*, *Basediomycota* and *Ascomycota* in the soil around *G. elata* Bl. *f. glauca* co-planted with JMG was higher, compared with the commercial strain CK (Fig. 7B). Second, Linear discriminant analysis Effect Size (LEfSe) analysis was used to test the difference in the relative abundance. Among these bacteria, only *p-Chloroflexi* contributed the most to the soil around *G. elata* Bl. *f. glauca* (Fig. 7C). Among the fungi, *Lecanicillium_fusisporum* contributed the most to the soil around *G. elata* Bl. *f. glauca* and *Bolbitiaceae* contributed the most to the soil around *G. elata* Bl. *f. glauca* tubers. (Fig. 7D). The results showed that there were significant differences in the relative abundance of bacteria and fungi between the two groups, with fungi contributing more significantly to the two groups, which confirmed that there are significant differences in the microbial composition and diversity between the two groups.

### PICRUSt functional prediction analysis of the soil around *G. elata* Bl. *f. glauca* co-planted with JMG

**Fig. 8:**
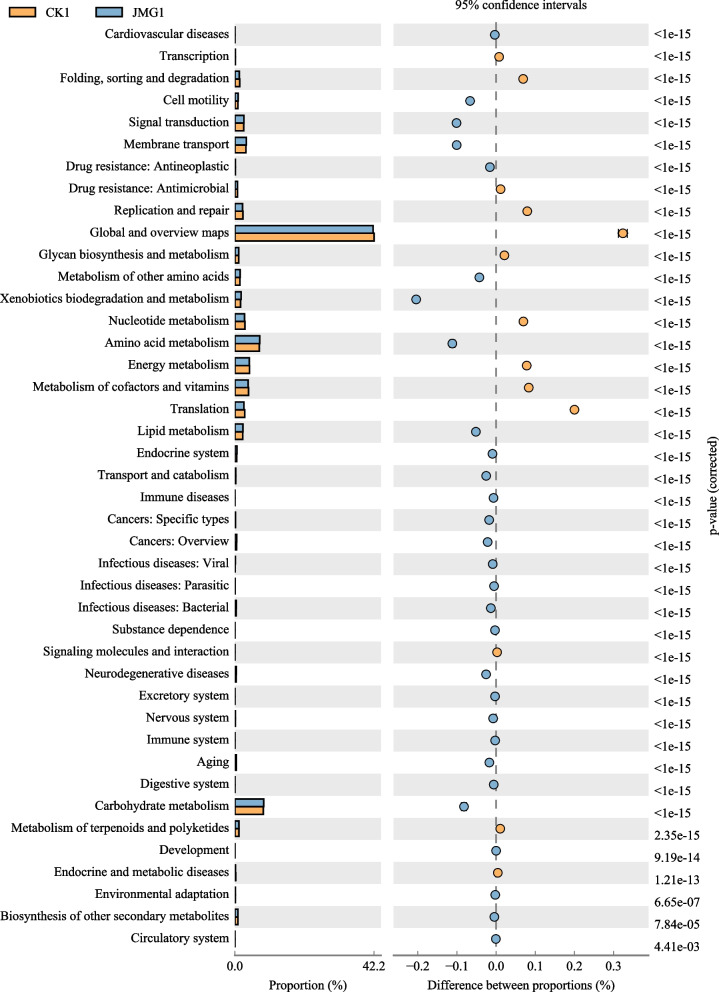
Difference analysis of KEGG metabolic pathways in soil bacteria around *Gastrodia elata* Bl. *f. glauca* between two symbiotic of it with JMG and CK. CK: The commercial *Armillaria* strain

The above research results demonstrated that JMG affects the composition and diversity of soil bacteria communities around *G. elata* Bl. *f. glauca*, and that there are significant differences between the two soil around *G. elata* Bl. *f. glauca* co-planted with JMG and the commercial strain CK. In order to further analyze the effect of microorganisms on their metabolic function, PICRUSt was used to predict and analyze the metabolic function of the soil bacteria community around *G. elata* Bl. *f. glauca*. The results showed that there were 42 secondary metabolic functions in the KEGG metabolic pathway of predicted genes at the secondary level, among which 17 secondary metabolic functions were different between the JMC and CK groups (Fig. 8). Among them, the copy number of seven secondary function predicted genes in the JMG group was greater compared with the CK group. They included these genes of Cell motilit, Signal transduction, Membrane transport, “Xenobiotics Biodegradation and Metabolism”, Amino acid metabolism, Lipid metabolism, other amino acid metabolism, etc. (Fig. 8). The copy number of 10 secondary metabolic function predicted genes in the JMG group was smaller, compared with the CK group. They included the genes of Transcription, Folding, sorting and degradation, Replication and repair, Global and overview maps, Glycan biosynthesis and metabolism, Nucleotide metabolism, Energy Metabolism, Metabolism of cofactors and vitamins, Translation, Carbohydrate metabolism, etc. (Fig. 8). The results showed that soil microorganisms around *G.elata* Bl. *f. glauca* under the symbiosis of it with different *Armillaria* strains had different metabolic functions.

In summary, JMG can improve the composition and diversity of the soil microbial community, increase the ability of *G. elata* Bl. *f. glauca* to absorb nutrients, and the metabolic function of soil microorganisms around it, which promotes its growth and development, thus increasing its yield. This may be one of the mechanisms for high yield of *G. elata* Bl. *f. glauca*.

## Discussions

The growth and development of *G. elata* Bl. are inseparable from the nutrition provided by *Armillaria*, and its isolation and identification have become a research hotspot [40]. A total of 15 *Armillaria* species were isolated and identified in China, among which eight endemic species were found to be widely distributed only in China [21]. However, not all *Armillaria* can provide nutrition for *G. elata* Bl. [41]. There are diverse symbioses between different *Armillaria* strains and *G. elata* Bl. [42], which has different effects on the yield of *G. elata* Bl. [23]. After multiple generations of *Armillaria*, its activity and ability to provide nutrients decreased, thus resulting in a decrease in *G. elata* Bl. production [27, 43, 44]. Therefore, it is necessary to isolate and identify more *Armillaria* strains, which are beneficial to the production of *G. elata* Bl.. In this study, five *Armillaria* isolates were obtained from the tubers and fruiting bodies of *Armillaria* officinalis in Changbai Mountain. Based on phylogenetic analysis, the amplified ITS regions of the five isolates were sequenced, conserved nucleotide regions are used for identifying different strains [45]. The five isolates were found to be more closely related to *A. gallica*, *A. sinapina* and *A. mellea*, which is consistent with previously reported *Armillaria* near Changbai Mountain and also confirms the M. strains that can be symbiotic with Amanita in previous studies [21]. These findings provided better Armillaria for improving the yield of endemic *G. elata* Bl. *f. glauca* cultivars in Changbai Mountain [46, 47].

*Armillaria* plays a crucial role in the growth of *G. elata* Bl. *f. glauca*. The strain quality directly affects the yield and quality of *G. elata* Bl. *f. glauca*, which are important indexes for screening high-quality *Armillaria*_._ Liu et al. compared the yield and effective components of *G. elata* Bl. co-planted with four different *Armillaria* strains in 2019. They found that the yield of *G. elata* Bl. co-planted with M1 strain is 1.905 times higher, compared with M3 one, and that the total amount of gastrodin and p-hydroxybenzyl alcohol in *G. elata* Bl. co-planted with the M1 strain is 2.4 times the standard of Pharmacopoeia, indicating that the yield and quality of *G. elata* Bl. co-planted with *Armillaria* are important indexes for screening high-quality *Armillaria* [41]. Additionally, the mycelial germination time, morphology and growth rate of different *Armillaria* stains are not the same, which directly affects their own biomass, and in turn exerts influence on the production of *Armillaria*, as well as the yield and quality of *G. elata* Bl.. This showed that they are also important indexes for screening high-quality *Armillaria* [48]. In this study, a multi-index evaluation method was adopted to comprehensively evaluate the strengths and weaknesses of *Armillaria*, which can help screen high-quality *Armillaria* strains more accurately, with more important guiding value for the production practice of *G. elata* Bl. *f. glauca.* Among the five *Armillaria* isolates, JMG has faster growth rates, and the yield and effective components of *G. elata* Bl. *f. glauca* co-planted with it are better, compared with the other isolates. Therefore, JMG is more suitable for forming a symbiosis with *G. elata* Bl. *f. glauca* in Changbai Mountain.

The vast majority of terrestrial plants on earth form a nutritional relationship of mycorrhizal symbiosis with fungi, which can provide plant bodies with various nutrients required for their normal growth and development [18]. Mycorrhiza can effectively improve the absorption of carbon, nitrogen, phosphorus, potassium, etc. by plants, thus promoting their growth and development [49]. Mycorrhizal fungi can directly provide varieties of nutrients and minerals required for the growth of orchid plants [50]. Mycorrhizal fungi such as *Cymbidiumhybridum* [51] and *D.nobile* [52] can not only markedly increase leaf and stem dry weights but also promote the absorption of N, P, K and other chemical elements by plants. Compared with commercial strains, JMG, promoted the absorption of various nutrition elements by *G. elata* Bl. *f. glauca*, which is one of the mechanisms by which this isolate increased the yield of *G. elata* Bl..

Soil microorganisms, an important component of soil, exist in the form of communities. And they are the drivers of the cycling and transformation of soil nutrients and organic matter [53], with an impact on soil quality and plant growth [54]. The species affecting the soil microbial community can be detected by investigating the diversity of soil microorganisms [55]. The growth and development of Orchidaceae are highly dependent on symbiotic mycorrhizal fungi. The impacts of symbiotic mycorrhizal fungi on the composition and diversity of soil microbial communities may lead to changes in the ecological environment of soil microgranisms around plants, thus improving the growth status and plant yield [56]. In this study, JMG could change the composition and diversity of soil microbial community around *G. elata* Bl. *f. glauca* [57]. For example, the relative abundance of beneficial fungi such as *Armillaria* in the soil around *G. elata* Bl. co-planted with JMG was higher, compared with commercial strains, while the relative abundance of *Cylindrosympodium* was lower. This may be one of the reasons why JMG isolate increased its yield by improving the soil microbial environment as well as the absorption and utilization of soil nutrients and disease resistance by *G. elata* Bl. *f. glauca* [58]. This result was consistent with that of the research on the effect of mycorrhizal microorganisms in some other plants on antagonizing soil pathogens and promoting beneficial soil fungi [59].

The metabolic function of microbial communities, such as the existence or deletion of functional genes, and their abundance, can be reliably predicted by PICRUSt analysis [60]. In this study, the functional genes of soil microorganisms around *G. elata* Bl. *f. glauca* co-planted with different *Armillaria* strains were predicted. Among them, compared with the CK group, the copy number of functional genes of amino acid and lipid metabolism and so on increased, while the copy number of functional genes of glycan biosynthesis and metabolism, nucleotide metabolism, cofactor and vitamin metabolism, energy metabolism, carbohydrate metabolism,etc. decreased. These changes in metabolic functions associated with plant growth [61] may also regulate the growth and development of *G. elata* Bl. *f. glauca* [62]. The increased copy number of functional genes of cell movement, signal transduction, membrane transport and so on may be correlated with the regulation of the nutrient absorption by *G. elata* Bl. *f. glauca* by JMG [63, 64]. The decreased copy number of functional genes of transcription, folding, classification and degradation, replication and repair and translation and so on may be correlated with the improvement in disease resistance in *G. elata* Bl. *f. glauca* by JMG [65–67]. These results indicated that JMG improves soil microbial metabolic functions of *G. elata* Bl. *f. glauca,* which differs from commercial strains. This may be another mechanism by which JMG increased its yield by improving soil microbial metabolic function, as well as soil nutrient absorption, utilization and disease resistance of *G. elata* Bl. *f. glauca*. At the same time, it will be a new research paradigm to see whether *Armillaria* can directly contribute to the enhancement of *G. elata* Bl. *f. glauca* production.

## Conclusions

In addition, some *Armillaria* strains can form a good symbiosis with *G. elata* Bl. *f. glauca*, which is closely related to the yield and the active component content of *G. elata* Bl. *f. glauca*. In this study, the five *Armillaria* strains were isolated and screened, and the most suitable symbiosis of *Armillaria* JMG with *G. elata* Bl. *f. glauca* was obtained. The following was found in this study: First, JMG could affect the nutrient absorption of *G. elata* Bl. *f. glauca*, especially it could increase the content of sodium and other chemical elements; second, JMG improved the microbial community in the soil around *G. elata* Bl. *f. glauca*, especially it increased the relative abundance of fungi such as *Armillaria*; finally, JMG also changed the metabolic function of microorganisms, especially Glycanbiosynthesis and metabolism, Nucleotide metabolism,etc. This preliminarily demonstrated how JMG can improve the yield and effective components of *G. elata* Bl. *f. glauca*. And further studies are certainly required for its molecular mechanism. These results provide new ideas for the research on the symbiosis of *Armillaria* and *G. elata* Bl. *f. glauca.*

## Materials and methods

### Isolation and identification of *Armillaria*

In this study, *G. elata* Bl. *f. glauca* tubers, rhizomorphs and fruiting bodies of *Armillaria* collected from Jingyu County, Baishan City, Jilin Province in 2019 were used to isolate and identify *Armillaria* strains. First, the tubers of *G.elata* Bl. *f. glauca*, and the rhizomorphs and fruiting bodies of Armillaria were washed with sterile water. After absorbing excess water with the sterile filter paper, *G.elata* Bl. *f. glauca* tubers were cut into 0.5 cm long sections on a sterile operation table, with each section of epidermis cut off (thickness 2 mm). Second, plates of corn-wheat bran medium(a solid medium prepared by sieving corn flour and boiling wheat bran with distilled water, filtering the solution with gauze, and adding agar powder) were inoculated one piece per plate. Meanwhile, take the rhizomorphs around the *G. elata* Bl. *f. glauca* tuber, rinse with sterile water 2–3 times, absorb the water on filter paper, pinch the two ends of the rhizomorphs, pull it off slowly and forcefully next to the flame of an alcohol lamp, cut the white mycelium 3-5 mm at the break with scissors, inoculated into potato dextrose agar (PDA) medium (culture 5 sections of mycelium per plate), then, 3–5 mm of thalli at the stipe and cap of *Armillaria* fruiting bodies were also inoculated into PDA medium plates, and each plate was inoculated with one piece. Third, the two plates were placed in a constant temperature incubator at 25 °C for 7–10 days in the dark. After the mycelium grew, the white mycelium was transferred to the plate of the corn flour and wheat bran medium for continuous culture. After the mycelium germinated, the tip of the mycelium was taken and inoculated onto the PDA medium for storage in the dark. Then, the *Armillaria* isolates were inoculated onto a new corn-wheat bran medium test tube, and a vernier caliper was used to measure the length of the mycelium every day. Then, the growth rate was calculated, and the morphology of the mycelium was observed and photographed through a microscope. Next, ITS genes of *Armillaria* was amplified by universal fungal-specific primers ITS 4 (F:5′-TCCTCCGCTTATTGATATGC-3′) and ITS 5 (R:5′-GGAAGTAAAAGTCGTAACAAGG-3′) [68]. After that, the amplified products were sent to Bioengineering (Shanghai) Co., Ltd. for sequencing. And these sequences were deposited in NCBI GenBank and compared with the sequences in GenBank by BLAST search. Phylogenetic analysis was performed by Mega7.0, and the prepared sequences were aligned by Clustal-W software [69, 70]. Finally, the phylogenetic tree was constructed by neighbor-joining (NJ) methods to analyze and compare the interspecific and intraspecific genetic distances of *Armillaria* sequences (the bootstrap test of 1000 times) [11, 45].

### Co-cultivation and harvest of *Armillaria* and *G. elata* Bl. *f. glauca*

The field experiment was conducted in the farmland base of Baishan Jingzhen Gastrodia Development Co., Ltd. Commercial strains and *G. elata* Bl. *f. glauca* seeds for cultivation were provided by Baishan Jingzhen Gastrodia Development Co., Ltd. The commercial strain, as the control check (CK) in this study, belongs to *A. gallica* and has been widely used for cultivating *G. elata* Bl.. As the main nutrient material, soybean straws were used for *Armillaria* bag culture medium, and the distribution ratio of raw materials was wheat bran(0–20%), corn(0–40%), and soybean straws with a particle diameter of 1–30 mm(40–100%). The materials were mixed evenly, followed by the addition of water to prepare a wet culture material with the final moisture content of 60–70. After autoclaving, it was put into a double-layer bag. The inner polypropylene bag measures 16.2 cm × 31 cm × 0.04 cm, and the outer polyethylene bag measures 16.5 cm × 34 cm × 0.04 cm. The five *Armillaria* isolates were inoculated into sterilized Armillaria bags, the bag mouth sealed, cultured at 22–25 °C for 15–20 min. and saved for later use. In May 2020, the field cultivation experiment was conducted, Several cultivation holes of *G. elata* Bl. *f. glauca* with a length of 50 cm, a width of 30 cm and a depth of 60 cm were first dug on the ground. Then, four tree sticks 6–9 cm in diameter and 40 cm in length, a fungus bag of different *Armillaria* isolates and 300 g *G. elata* Bl. *f. glauca* seeds were simultaneously placed in the cultivation holes. With each *Armillaria* isolate as a treatment unit, 10 cultivation holes were included in each treatment unit. In November of the same year, *G. elata* Bl. *f. glauca* in the experimental site was harvested, with the performance of random harvest for five holes per treatment unit. The fresh *G. elata* Bl. *f. glauca* was weighed and the data recorded.

### Determination of effective components of *G. elata* Bl. *f. glauca* co-planted with different *Armillaria* isolates

Standard samples of gastrodin, p-hydroxybenzyl alcohol, balisonoside A, parisenoside B, balisonoside C and balisonoside E were purchased from Shanghai Yuanye Biotechnology Co., Ltd. The active ingredients were determined separately from *G. elata* Bl. *f. glauca* co-cultivated with different isolates of *Armillaria*. First wash and dry the sample with water, and it was steamed for 30 min, dried in an oven at 60 °C to a constant weight and finally made into powder. Firstly, 4 mg of each of gastrodin standard, p-hydroxybenzyl alcohol standard, balisonoside A standard, parisenoside B standard, balisonoside C standard and balisonoside E standard were weighed and fixed in a 5 mL volumetric flask with acetonitrile-water (3:97) mobile phase to make 0.800 mg-mL-1 of the control solution. The standards of gastrodin, p-hydroxybenzyl alcohol, balisonoside A, balisonoside B, balisonoside C and balisonoside E were precisely aspirated according to 2:1:2:2:1:2, and prepared as a mixed control solution containing 20 μg, 10 μg, 20 μg, 20 μg, 10 μg and 20 μg per 1 mL. The concentrations of the mixed controls were increased in a sequential gradient. The standard solutions were determined by high performance liquid chromatography (HPLC), the peak areas were calculated and the standard curves were plotted using the concentration (μg-mL-1) as the horizontal coordinate and the peak area as the vertical coordinate. Then, 2.000 g *G. elata* Bl. *f. glauca* powder was accurately weighed, and put in a conical flask with a stopper, followed by the addition of 50 mL of 50% dilute ethanol and weighed. The powder solution was extracted for 30 min (power 120 W, frequency 40 kHz) at room temperature and weighed after cooling, and dilute ethanol was used to make up for the lost mass. 15 mL solution was absorbed into the centrifugal tube with a liquid transferring gun and centrifuged at 3000 r/min for 20 min. And 10 mL supernatant was precisely absorbed into the evaporating dish, concentrated at 60 °C until no alcohol flavor, followed by the addition of acetonitrile-water (3:97) mixture to dissolve the residue. Next, the dissolved residue was transferred to a 25 mL volumetric flask, with its volume made up to scale with acetonitrile-water (3:97) mixture, shaken well, passed through a 0.220 μ m filter membrane and set aside. After that, the content of each active component was measured by HPLC, with a mobile phase of acetonitrile (A) and 0.05% phosphoric acid solution (B) (3:97). The detected wavelength was 220 nm, with a column temperature of 35 °C. The volume flow was 1.0 mL/min, with gradients of 98 to 90% B (0–10 min), 90 to 88% B (10–15 min), 88 to 82% B (15–25 min), 82% B (25–40 min), 82 to 5% B (40–42 min), 60% B (42–47 min), 40% B (47–52 min), and 97% B (52–62 min). The injection volume was 20 μ L, and the Century SIL C18 column (250 mm × 4.6 mm,5 μ m) was adopted. The content of each active ingredient in the sample was calculated from the standard curve.

### Analysis of absorption Laws of nutrition elements of *G. elata* Bl. *f. glauca* co-planted with JMG

0.5 g tubers of *G. elata* Bl. *f. glauca* co-planted with each *Armillaria* isolate and its surrounding soil were weighed with an accuracy of 0.001 g, respectively, followed by the addition of 5.0 mL HNO3 and 1.0 mL H2O2 to the microwave digestion tank, and mixed well. And the tank lid was tightened. Then, the mixture was digested at 120 °C for 4 h. After the digestion, it was cooled and taken out. Next, the tank lid was slowly opened, the acid injected to about 0.5 mL, and then1% nitric acid was used to make up the volume to 50 mL, shaken well for later use. 1% HNO3 was utilized as the blank control solution. The processed samples were measured by inductively coupled plasma mass spectrometry (NexIONTM350X)--0.60 V; Atomization gas flow(0.90 L/min); Cooling gas flow(1.50 L/min); Auxiliary gas flow(0.80 L/min).

### Analysis of Interweek variations in soil microbial community diversity around *G. elata* Bl. *f. glauca* co-planted with JMG

The commercial strain *Armillaria* and Changbaishan *G. elata* Bl. *f. glauca* used as the CK samples were provided by Baishan Jingzhen Gastrodia Development Co., Ltd. JMG samples are the dominant strains screened in our previous research work. Each *Armillaria* isolate and *G. elata* Bl. *f. glauca* were weighed and take a uniform sample of 5–10 g of soil in the same position around the circumference, three times for each sample, and put in a sterile zip-lock bag. After natural air-drying, impurities such as animal and plant residues and gravel were removed, with large samples smashed. After passing through a 2 mm sieve, the samples were aliquoted into 2 mL EP tubes, with a soil content of 0.25–0.5 g in each tube, and the sample volume was guaranteed to be 1-2 g. After aliquoting, the samples were stored in liquid nitrogen, with the collection of three replicates for each soil treatment.

Total DNA extracted from soil served as a template to amplify bacterial 16 s rDNA and fungal ITS, respectively. The PCR products were purified, quantified and homogenized by bacteria, with 338F (5 cm-ACTCCTACGGGAGGCAGCA-3′) and 806R (5 mm-GGACTACHVGGGTWTCTAA Tmer3’) as universal primers, and by fungi, with ITS 1 (F: 5′-CTTGGTCATTTAGAGGAAGTAA-3′) and ITS 2 (R: 5′-GCTGCGTTCTTCATCGATGC-3′) as universal primers. PCR products were sequenced by Illumina HiSeq 2500 and USEARCH software (version 10.0) [71] was used to cluster high-quality sequence data with a 97% quality control level, with the adoption of Venn diagrams for displaying the number of common and unique ones among samples [72]. The RDP classifier Bayesian model algorithm was adopted to perform the species- type classification and sequence annotation for each sample in the species sequence information database. The bacterial 16S rRNA Silva database (Release132, http://www.arb-silva.de), and the Unite fungal ITS one (Release 8.0, https://unite.ut.ee/) were utilized. QIIME software was used to generate species abundance tables at different taxonomic levels, followed by the adoption of R language tools to draw community structure diagrams and species clustering heat maps of the samples at each taxonomic level, and Python language tools were utilized to draw the phylogenetic diagram at the taxonomic level of the genus [73]. Finally, α-diversity indexes of the samples were evaluated by QIIME2 software, and the Ace, Chao1, Shannon and Simpson indexes were calculated, respectively [74]. The curves of the dilution, rank abundance and species accumulation reflected by Shannon diversity indexes were plotted by Mothur software and R language tools [75]. The Unweighted paired average method (UPGMA) was adopted to cluster the samples, and plot their heatmap, and then the similarity of species composition among them was judged through R language tools [76]. ANOVA and Linear discriminant analysis Effect Size (LEfSe) [77] were applied to the differential analysis between groups. The PICRUSt2 [78] software was adopted for annotating the species in feature sequences to be predicted and the existing phylogenetic tree. And the IMG microbial genome database was used for functional information output to infer the composition of functional genes in the samples, and in turn analyze functional differences between different samples or groups.

## Statistical analysis

Statistical analysis of all data was performed on significance at a significance level of 0.05 by one-way ANOVA and LSD and Duncan’s test.

## Supplementary Information

**Additional file 1. Table S1.** Growth characteristics of five *Armillaria* isolates under the same culture conditions.

**Additional file 2. Table S2.** Homology alignment of five *Armillaria* isolates in GenBank.

**Additional file 3. Table S3.** Yield of different *Armillaria* isolates co-planted with *Gastrodia elata* Bl. *f. glauca.*

**Additional file 4. Table S4.** Element contents and enrichment factors of *Gastrodia elata* Bl. *f. glauca* and soil under different treatments.

**Additional file 5.** **Table S5.** GenBank accession numbers of different species.

**Additional file 6.** **Table S6.** Table of fungal and bacterial alpha diversity indices.

**Additional file 7.** **Fig. S1.** Detection of 6 sample solutions by high performance liquid chromatography.

## Data Availability

The sequence data generated in this study can be obtained in NCBI database through login numbers MW404994(Armillaria gallica isolate 1 internal transcribed spacer 1, partial se - Nucleotide - NCBI (nih.gov)), MW404995(Armillaria sinapina isolate 2 small subunit ribosomal RNA gene, partia - Nucleotide - NCBI (nih.gov)), MW404996(Armillaria sinapina isolate 3 small subunit ribosomal RNA gene, partia - Nucleotide - NCBI (nih.gov)), MW404997(Armillaria sinapina isolate 4 internal transcribed spacer 1, partial s - Nucleotide - NCBI (nih.gov)) and MW404998(Armillaria sinapina isolate 5 internal transcribed spacer 1, partial s - Nucleotide - NCBI (nih.gov)). All data analyzed during this study were included in this manuscript and its additional files.
