# Supplementary material for: An exploration of mechanism of high quality and yield of Gastrodia elata Bl. f. glauca by the isolation, identification and evaluation of Armillaria
Source: BMC Plant Biol. 2022 Dec 30;22:621. doi: 10.1186/s12870-022-04007-8 (PMC9801631; doi:10.1186/s12870-022-04007-8)
Supplement: Supplementary file 7 — Additional file 7. Fig. S1. Detection of 6 sample solutions by high performance liquid chromatography. [file 12870_2022_4007_MOESM7_ESM.pptx]

## Slide 1
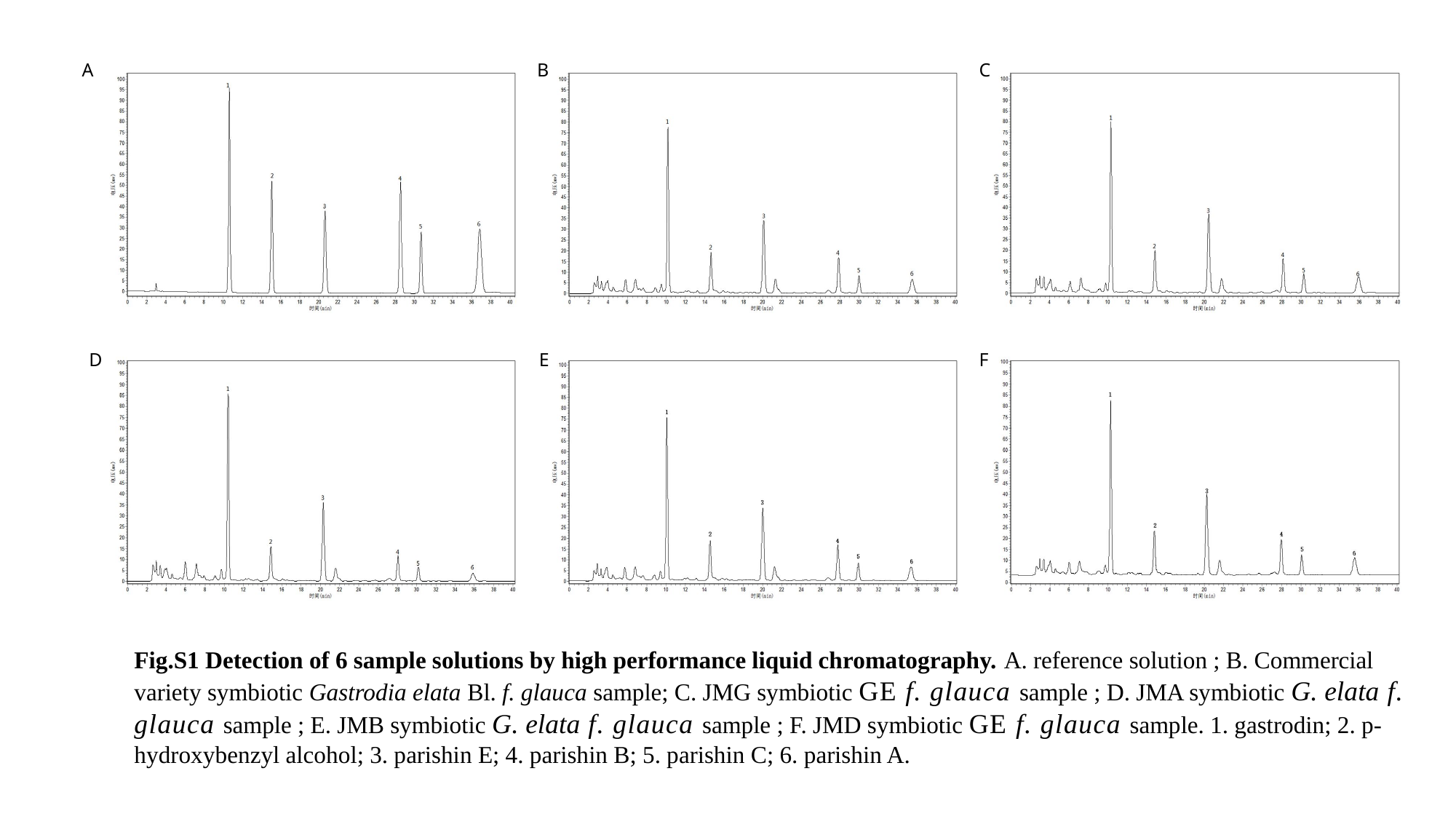

C
B
A
F
E
D
Fig.S1 Detection of 6 sample solutions by high performance liquid chromatography. A. reference solution ; B. Commercial variety symbiotic Gastrodia elata Bl. f. glauca sample; C. JMG symbiotic GE f. glauca sample ; D. JMA symbiotic G. elata f. glauca sample ; E. JMB symbiotic G. elata f. glauca sample ; F. JMD symbiotic GE f. glauca sample. 1. gastrodin; 2. p-hydroxybenzyl alcohol; 3. parishin E; 4. parishin B; 5. parishin C; 6. parishin A.
